# The Cultural Epigenesis of Gender-Based Violence in Cambodia: Local and Buddhist Perspectives

**DOI:** 10.1007/s11013-017-9563-6

**Published:** 2018-01-16

**Authors:** Maurice Eisenbruch

**Affiliations:** 1 Department of Psychiatry, School of Clinical Sciences at Monash Health, Monash University, Clayton, Australia; 2 Royal University of Phnom Penh, Phnom Penh, Cambodia

**Keywords:** Gender-based violence, Violence against women, Cambodia, Cultural epigenesis, Ethnography, Buddhism, Violence

## Abstract

Almost one in four women in Cambodia is a victim of physical, emotional or sexual violence. The study aims to provide a comprehensive understanding of the ways in which Cambodians see its causes and effects and to identify and analyse the cultural forces that underpin and shape its landscape. An ethnographic study was carried out with 102 perpetrators and survivors of emotional, physical and sexual violence against women and 228 key informants from the Buddhist and healing sectors. Their views and experiences of it were recorded—the popular idioms expressed and the symptoms of distress experienced by survivors and perpetrators. From these results, the eight cultural forces, or cultural attractors, that are seen to propel a person to violence were identified. Violence stemmed from blighted endowment, or ‘bad building’ (*sɑmnaaŋ mɨn lʔɑɑ*) determined by deeds in a previous life (*kam*). Children with a vicious character (*kmeeŋ kaac* or *doṣa*-*carita*) might grow to be abusers, and particular birthmarks on boys were thought to be portents. *Krʊəh*, or mishap, especially when a female’s horoscope predicted a zodiac house on the descent (*riesəy*), explained vulnerability to violence and its timing. Astrological incompatibility (*kuu kam*) was a risk factor. Lust, anger and ignorance, the ‘Triple Poison’, fuelled it. ‘Entering the road to ruin’ (*apāyamuk*), including alcohol abuse, womanising and gambling, triggered it. Confusion and loss of judgement (*mohā*) led to moral blindness (*mo baŋ*). These were the eight cultural attractors that shaped the landscape of violence against women. The cultural epigenesis of violence against women in Cambodia is an insight which can be used to build culturally responsive interventions and strengthen the primary prevention of violence against women. An understanding of the epigenesis of violence could strengthen the primary prevention of violence against women.

## Background

The most recent evidence confirms that gender-based violence (GBV) against women around the world is culturally constructed, shaped, and in some cases prevented. Examples of such studies include the Arab world (Standish 2014), Bangladesh (Fattah and Camellia 2017; Chowdhury 2014), Bosnia (Muftić and Bouffard 2008), China (Tang, Wong and Cheung 2002), India and China (Taylor, Xia and Do 2017), Myanmar (Norsworthy and Khuankaew 2004), Nepal (Puri, Shah and Tamang 2010), Nigeria (Oladepo, Yusuf and Arul 2011), Pakistan (Sadiq 2017), Rwanda (Zraly and Nyirazinyoye 2010), Senegal (Bop 2010), Thailand (Ezard 2014; Han and Resurreccion 2008), Turkey (Ozcakir et al. 2008) and Vietnam (Krause et al., 2015). Despite the undeniable role of culture, a role that is universally acknowledged, seldom does it find an easy home in the discourse on GBV.

### Cambodia

The aim of this article is to discover how the ‘insider’ views the causes and effects of GBV in Cambodia. In so doing, my focus is not so much on violence against women across the board, such as violence against female household heads who are the weakest parties in land-related human rights abuses (Kent 2011) or of female human rights defenders, but on intimate partner violence. My central theme is to reveal the perceived causes of GBV in Cambodia. While government rhetoric tends to focus on alcohol and poverty, this article looks more closely at the cultural bedrock of these undeniably powerful factors so that they can be better understood and used in the national efforts to prevent GBV.

Cambodia has consistently appeared at or near the top of all recent rankings on corruption and impunity (Transparency International 2016) and remains among the most violent countries on the planet. There are escalating rates of murder, robbery, and banditry (Broadhurst 2002). Violence against women continues to be serious (Brereton et al. 2009; Fulu et al. 2015) as does the spate of acid attacks to maim and isolate them (Waldron et al. 2014) and which, despite journalistic reports to the contrary, have persisted since the Law on Regulating Concentrate Acid in 2012 was passed. Among young people, substance abuse is also a cause of violence against women (Yi et al. 2016). At a public level, multinationals make microcredit widely available with scant regard for the consequences in threatening the livelihoods of the economically most vulnerable and precipitating further erosion of family safety (Ovesen and Trankell 2014). Intimidation is ubiquitous, and the incidences of forced evictions, displacements and human rights violations continue to rise (Brickell 2014). Cambodia’s leaders flaunt their impunity shamelessly in public (Khuon and Willemyns 2015), human rights offenders routinely escape justice (ADHOC et al. 1999) and civil society organisations have appealed for a stop what they call ‘the culture of impunity and violence’ (LICADHO 2012).

#### Violence, Culture and Buddhism

This atmosphere of violence might seem surprising in a country where Buddhism is the national religion, and where, from time to time, Cambodian Buddhism has connected with radical religious narratives for peace. Monks and female devotees have been involved in ‘engaged Buddhism’—the contemporary movement of nonviolent social and political activism found throughout the Buddhist world (King 2005)—applying the insights from *dhamma* teachings to circumstances of social injustice and structural violence (Rothberg 1997). Paradoxically, the Khmer Rouge had its origins in the Buddhist nationalism of the 1940s, and Cambodia was among the countries where, in the post-colonialist phase, there were efforts to merge Buddhism with socialist ideals (Ladwig and Shields 2014).

In Cambodia, the annual *dhammayietra* or ‘pilgrimage’ marches during the 1990 s aimed at extinguishing the flames of war, and women’s networks joined Buddhist leaders to mobilise a mass peace movement (McGrew et al. 2004), while Cambodians wrote about eliminating the suffering of their people through Buddhism (Thach 1993). More recently, monks have taken action to combat radical or extreme violence in Cambodia, but these activities have not borne fruit in national understandings of violence or strategies to deal with it (Kent 2006).

We have to ask the difficult question whether Cambodian Buddhism contributes to conflict and gender inequality and therefore to GBV. Johan Galtung showed how ‘cultural violence’, the aspects of culture that can be used to justify direct and structural violence, has an opposite in ‘cultural peace’, meaning aspects of a culture that serve to justify direct and structural peace (Galtung 1990). In Cambodia, Hughes (2001) says the ‘reverence for Buddhist teachings’ is a useful basis for new approaches to conflict management, while Gelleman (2007) observes how Cambodian monks mediate conflict in ways that echo Lederach’s model (1995) of ‘elicitive conflict transformation’.

In an echo of Galtung, it is true that ‘Religious institutions can be vital allies in shifting norms around violence but equally can be responsible for defending violations of women and girls’ rights’ (Action Aid and Moosa 2012). The Buddhist temple provides some opportunities for abused women to seek refuge and recovery (Kent 2011). If they are to do more, by shifting norms of violence, the best way for this to happen is by understanding the good they can do and encouraging them to do so, rather than by criticising Buddhism as a conservative force for gender inequality. Unfortunately, it is also true, as I am discovering in recent work, that a minority of monks (painted by the government as ‘a few bad apples’) are themselves perpetrators of sexual abuse against girls and boys; fortunately, the vast majority of monks (and the female devotees who live in the temples and serve them) defend the rights of women, and some ‘engaged Buddhists’ do so very actively indeed.

#### Violence Against Women in Cambodia

The levels of violence against women in Cambodia are alarming (Fulu et al. 2015) and often not reported (UN Women 2014). The regional UN Multi-Country Study on Men and Violence survey (Fulu et al. 2013b) found that 32.8% of men in Cambodia reported perpetrating physical and/or sexual violence against an intimate partner in their lifetime, and one in five men reported raping a woman or girl, one of the highest recorded rates in the Asia-Pacific region (Fulu et al. 2013a:9). Papua New Guinea-Bougainville, Indonesia-Papua and Cambodia have the highest rates of gang rape (*bauk* in Khmer) in the region. Gang rape in Cambodia is reported to be linked with aspects of the culture related to sexual entitlement (Wilkinson, Bearup and Soprach 2005) and prompted by viewing gang rape pornography (Farley et al. 2012). Fulu and Miedema (2015) point out that the situation has been made both worse and better by the effects of globalisation: economic development has meant that women have flocked to the cities in search of work, a situation that prompts more GBV; at the same time, the heightened awareness and activism arising from the global women’s rights discourse has brought GBV to the fore as a social issue of the highest importance.

There is also an intergenerational loop of violence. More than half of all children in Cambodia also experience violence, often based on gender (Ministry of Women’s Affairs et al. 2013). UNICEF data show the devastating effects of GBV on girls, who can grow to self-harm, suffer severe mental distress and, ultimately, endure intimate partner violence (Fang and UNICEF 2015).

Literary texts such as the ‘woman’s code’ (*cbap srəy*) reinforce domestic abuse as acceptable (Surtees 2003). Chandler (1984) depicts the *cbap*, as gnomic, normative poems popular in pre-colonial Cambodian society and which, unlike chronicles or inscriptions, depicted the activity of the entire society rather than that of just the elites, and which helped people confront the harsh realities of everyday family life. Brickell (2011) highlights the different cultural discourses that men and women draw on to explain the persistence of inequality, which she terms a ‘stubborn stain’ on development. The usual suspects—cultural condoning of alcohol abuse, sex outside marriage and challenges to traditional male masculinity—have been well documented (GADC 2010). It has been suggested that men exploit cultural hegemony in various ways, such as by blaming alcohol, or prioritising the harmony of the home over the well-being of women. Brickell and Garrett (2015) used collective storytelling in participatory video workshops to explore the hegemonic norms, and they found that the primary community perceptions of the ‘cause’ was alcohol.

This focus can be broadened. Trudy Jacobsen (2012) notes the conflict for men who, on the one hand, are expected to ‘act out the manhood script’ and ‘correct’ their erring wives and children—through violence if necessary—and, on the other, to adopt ‘the alternative model of a man who is self-controlled’. According to Jacobsen, in the past it was the norm for men and women to have complementary roles that were valued equally, and only after the colonial era was this view replaced by the assertion that domestic work is women’s work and less important than men’s.

If Jacobson is right, then cultural traditions have an in-built sanction against GBV which needs to be better understood. Jacobsen’s findings pose difficulties for the western standard bearer who challenges Cambodian hegemonic notions of masculinity, showing how the Cambodian past, including its assigned gender roles, is bound up with national identity and the view that it is ‘culturally essential that the manhood script remain static’ (Ford and Lyons 2012).

Work (2014) says that youth who lack the capacity to achieve the ‘traditional indicators’ of successful masculinity can instead engage in violent behaviour as ‘an avenue for enacting models of powerful manhood’. Work (2014) and Jacobsen (2012) both depict the tension between two styles: the ‘good man’ who should be hardworking and show compassion towards others, and the ‘successful man’, who is a bit like the ancient kings and, ‘favoured by the hegemonic masculinity of the neoliberal global order’, sexually and personally aggressive in a context where ‘masculine performance involves conquering women, earning money, and spoiling for a fight’.

These studies are springboards for further elucidating the cultural construction of violence against women—how their causes and consequences are perceived, and how, in this tapestry of violence, the warp of the social-economic-political yarn is interwoven with the weft of cultural meaning. What is at stake is how these cultural insights are used, or bypassed, in developing new theories and approaches to change.

To provide a comprehensive understanding of the ways in which Cambodians see the causes and effects of violence against women in Cambodia and to expose and analyse the cultural forces that underpin and shape its landscape, it is important that everyone working to prevent or mitigate violence in Cambodia be familiar with its local idiom, and it is hoped that the detailed case studies in this research will take us closer to the local texture of violence.

The article sets out the epigenesis of violence against women. I document the local understanding by perpetrators and survivors and their families, as well as monks and healers, of the causes and effects of intimate partner violence, and include the Buddhist and folk stories they use to describe it. I explore the popular idioms expressed by a range of people to depict violence as they know it and delineate the local symptom patterns of mental and physical distress experienced by survivors and perpetrators.

The article moves through eight ‘cultural attractors’, demonstrating how each works in propelling a person to violence. First, I consider how violence is seen to stem from blighted endowment, or ‘bad building’ (*sɑmnaaŋ mɨn lʔɑɑ*), determined by deeds in a previous life (*kam*). Then I discuss the perceived role of vicious character in early life (*kmeeŋ kaac* or *doṣa*-*carita*) that might lead to a person becoming an abuser, and particular birthmarks on boys which are thought to be portents. Next, I discuss the powerful local concept of *krʊəh*, or mishap, especially when a female’s horoscope predicted a zodiac house on the descent (*riesəy*), as explaining vulnerability to violence and its timing. Given the importance of marital harmony, I then consider astrological incompatibility (*kuu kam*) as a risk factor and what people seem to feel can be done about it. I then present a particularly Buddhist perception that GBV is fuelled by lust, anger and ignorance, the ‘Triple Poison’. The usual suspects of alcohol and poverty are considered in the next cultural attractor, called ‘entering the road to ruin’ (*apāyamuk*). The two final cultural attractors I present have to do with the mental state of a perpetrator, depicted here as confusion and loss of judgement (*mohā*), followed by moral blindness (*mo baŋ*). In an epigenetic formulation, I show how the ‘cultural attractors’ make up the landscape of GBV.

## Method

The author is a Khmer-speaking medical anthropologist and transcultural psychiatrist. He was supported by Cambodian research assistants—Chou Sam Ath in Cambodia who carried out many of the fieldwork interviews, and Phally Chun and Thel Thong in Melbourne who assisted with the data analysis and interpretation. The research is based on the author’s ethnographic and clinical research since 1990 in Cambodia, with a focus since 2004 on violence. From the start, the fieldwork involved people affected by violence. Ethics approval was obtained from the National Ethics Committee for Health Research (NECHR) in Cambodia. The researchers were not involved in the clinical care of those affected. All fieldwork was conducted in Khmer and translated into English.

### Conceptual View of the Epigenesis of GBV

In advancing this deeper exploration, I am inspired by two conceptual advances. The first is the breakthrough by John Wilson in viewing the effects of exposure to violence as altering the epigenesis of identity in the life cycle within culturally shaped parameters (Wilson 2007). The second is Iddo Tavory, Jablonka and Ginsburg (2012) metaphoric depiction of culture and epigenesis. Tavory explains that the social landscape is underpinned by a network of ‘cultural attractors’, by which he means ‘mutually reinforcing mechanisms that lead an individual to a shared outcome in a landscape underpinned by a series of interacting elements’. These advances are built on Chas Waddington’s pioneering work on epigenetic landscapes (Waddington 1957).

### Participant Selection

An ethnographic study was carried out with 102 perpetrators and survivors of gender-based violence. Most of the cases (n = 79) involved men committing abuse against women (each case could have featured more than one form of violence—25 physical, 20 sexual, 43 emotional and 35 psychological). In a further 13 cases, women were perpetrators (3 physical, 8 emotional and 9 psychological). Three cases involved abuse being perpetrated by a man and woman together. The women in the study were aged between 18 and 52, and the men were slightly older. Cases were from Kampong Cham, Kampong Chhnang, Kampong Speu, Kandal, Siem Reap and Takeo provinces and in Phnom Penh. This sample was drawn from a larger study of direct and public violence, which included other forms of family violence involving a parent or other family member, but these were excluded from this report as being outside the definition of intimate partner violence.

The fieldwork involved 54 key informants, many of whom were directly familiar with the cases, comprising 17 monks, 4 Buddhist ritual officiants, 1 male and 5 female Buddhist devotees, 15 mediums, 9 traditional healers and 3 traditional birth attendants. Some of these were known to us as knowledgeable and morally committed ‘engaged Buddhists’ who were working in effect as ‘Buddhist psychotherapists’ and ‘Buddhist traumatologists’. Other participants, such as village heads and people in the neighbourhood, were also included.

Through participant observation, we observed abusers and survivors consulting monks and healers and documented the details of ritual interventions carried out. We followed up these families in their homes and on return visits for further ritual interventions.

### Fields of Exploration

Informants’ views of impunity and violence were documented. Their lexicon was recorded, including the use of images and metaphor, and popular folk legends were probed for echoes of violence. We explored the way that communities, using a combination of Buddhist and rights-based notions, made sense of violence and we recorded the rituals performed and the legends recited by monks for survivors and perpetrators. Sampling continued until we reached the point of data saturation. This method encouraged the informants, once they felt more trusting, to share their views rather than confining themselves to the rights-based views they thought we wanted to hear.

We documented reports in Khmer- and English-language newspapers and social media and sometimes followed up with fieldwork visits. Some informants were shown pictures of *yantra*—magical designs inscribed by monks or traditional healers on cloth or metal and carried as protection from natural and supernatural harm—used to protect people against violence, or DVDs or videos of ‘moral blindness’ and ‘the road to ruin’, and their reactions to them and how they were connected with their personal experiences of violence, were recorded.

### Data Analysis

Samples of recordings were translated and back-translated. We analysed the cultural idioms of violence, examined how informants expressed their states of mind to see how meaning was drawn from context, and paid careful attention to their cultural registers and use of Khmer popular cultural references. Comparisons were made between formal and informal Buddhist themes.

### Derivation of Themes

Using qualitative data techniques, we analysed manifestations of violence against women as assessed in particular cultural idioms such as bodily ‘undergoings’ and associated feelings; and public symptoms of feelings that communicate affect to others. We also analysed larger-scale understandings of self and society as locally understood: what is being threatened, where is the violence felt? In what kinds of binds are survivors and perpetrators? What kinds of social relations organise local responses—such as sympathies, surveillance, stress, succour—to survivors and perpetrators? What new conditions have been introduced into people’s lives? What is the history of this violence—where did it happen first and how did it spread? Who is susceptible?

We analysed this language of violence through thematic and content analysis and systematically organised the data into a structured format. Identifying root concepts such as anger, jealousy and impunity, we noted the collocations, the sequences of words or terms that co-occurred and the Pali or Sanskrit roots from which the more ancient terms originated. In semantic analysis, we analysed the literal and figurative language of violence and, in pragmatic analysis, analysed how the meaning is derived from context and the attributions of each of the terms they used. We also took the register and influence of popular Khmer culture into consideration.

Being able to read both the idiomatic and literal English bracketed with the Khmer terms is essential for readers to be able to use the material in developing training vignettes, for example, or for use in the field. Khmer terms are spelled using Huffman’s adaptation of the IPA phonetic transcription (Huffman, Lambert and Im 1970), rather than transliteration, to help non-speakers of Khmer more easily and consistently pronounce the terms.

The danger of stereotyping and stigmatising people as ‘perpetrators’ or ‘abusers’ and ‘survivors’ is acknowledged, but for the sake of brevity use of these terms is unavoidable. Names of informants have been changed and their locations de-identified except when the events reported are already widely in the public domain.

## Results

**Fig. 1:**
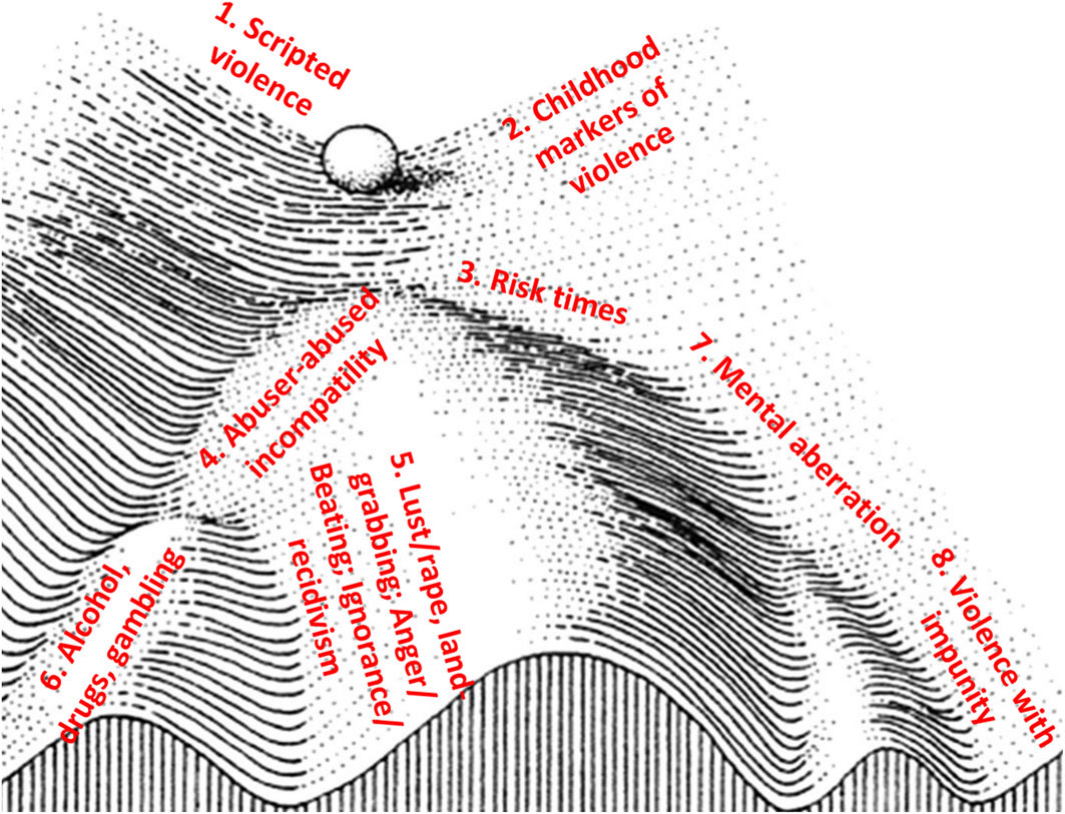
Epigenetic landscape of violence, showing its aetiology and features

The results revealed eight ‘cultural attractors’, which for simplicity are set out as eight clusters. Inspired by Waddington and his brilliant exposition on epigenetic landscapes, the sequence set out here presents the reader with a Waddingtonian landscape that reveals the cultural drivers of gender-based violence in Cambodia (Fig. 1). The ‘violence history’ of the person, abuser or abused, is represented by a ball rolling down a landscape with hills and branching valleys. It starts with scripted violence, which is the endowment from birth of the perpetrator or victim, and continues through successive manifestations such as the childhood markers of violence or the times of risk and vulnerability in the life of the perpetrator or survivor, or the development of feelings of impunity, which permits unbridled violence to erupt shamelessly. The life experience of survivor or perpetrator, represented as the sphere at the top of the landscape, is shaped by the way it traverses the landscape. Each person can be pulled along and descends along one or more valleys.

### Scripted Violence-*Sɑmnaaŋ mɨn lʔɑɑ*

Gender-based violence was sometimes thought to be scripted into the life of a perpetrator or survivor, built on bad foundations (s*ɑmnaaŋ mɨn lʔɑɑ*)—the first ‘cultural attractor’—and scripted according to their deeds in a previous life (*kam*).

Heng and Kolap’s marriage had deteriorated when she became pregnant. Heng’s family had never accepted her because of her family’s poverty. Heng, under his family’s influence, tried to kill Kolap and their unborn child by cutting the hammock on which she sat. Without waiting for the baby to be born, Kolap left him and lived with her mother. Looking back, Kolap realised that she had ‘bad building’, unable to shake off the ‘thick *karma*’ that had made generations of her family poor, illiterate and prone to violence.

This could be an example of an imprinted intergenerational history of violence.

Chann was an alcoholic wife-beater. His parents had ‘married’ in the wake of the Khmer Rouge regime without being able to consult an astrologer, and his father became a heavy drinker who womanised and abused his wife. An NGO worker, knowing Chann’s parents’ history, told me that the abuser had lived with a ‘family line of violence that was imprinted from generation to generation’. Chann’s brain had ‘photographed the violence’, he said.

The ‘bad building’ was founded on violence and led to a re-enactment of it. Some abusers said they had never broken their links with the stain of violence inflicted on them in a previous life, which they said they remembered (*cam ciet*). One abusive husband said he was a reincarnated tortured person, his wrists bound with hemp before execution, and he showed me the scars he had had since birth.

Blighted endowment is not just about violence affecting individuals. Older people recounted Khmer phrases from the millenarian ‘Buddha Predictions’:

A group of three women in Siem Reap told me how they, and many others in the region, were abused by their husbands. They attributed this upsurge to the Three Vast Plains of illness, hunger and war. In a panic, and fearing that more troubles were on the way, they frantically handwrote copies of a millenarian *yantra* of *Buddh Damnāy* and circulated them to those they thought to be threatened by mass violence.

In describing the world swamped by bloodshed, including through violence against women, some used expressions such as, ‘Blood is close to the elephant’s belly’ (*cʰiem* daap *pʊəh dɑmrəy*), meaning that the world would be soon be inundated by bloodshed, some of which could be attributed to men’s violence against women.

### Childhood Markers of Violence-*Carita*

Precocious boys born with the stigmata of lust (*kmeeŋ kʰəl*) were thought to grow into sexual predators of women. The monks said these boys were examples of the first ‘Triple Poison’ (*rāga*-*carita*). Children of vicious character were *kmeeŋ kaac,* the second ‘Triple Poison’ (*doṣa*-*carita*). Such boys grew to be abusers. Girls with vicious characters grew to become ‘wild forest hens’ (*moan prey*), step-wives or lovers who would enter the ‘pen’, that is, the family compound, and fight ‘dirty’, using their beaks and claws to exert control and viciously wound the wife. Boys who were naïve and uncurious about the world were literally ‘in the dark’ (*ŋɔŋɨt lŋɔǝŋ*), and prone to become abusers. Monks classified them as *mohā*-*carita*, which they linked with the third ‘Triple Poison’.

A husband’s disinclination to listen was also a marker of violence. Abused wives said their words ‘went in one ear and out the other’ (*cool trɑciək muəy, cəɲ trɑciək muəy*), or using the Khmer expression, ‘Like pouring water on the duck’s head’ (*dooc cak tɨk* *ləə kbaal tie*), or the English one, ‘Like water off a duck’s back’. Their husbands were impervious to counselling and continued to get drunk and beat them.

Girls were said to be imbued with a character that foretold their conduct as women. A girl who has learned the traditional values of being ‘good and straight like a fresh sarong that is new and unsoiled and folded with sharp creases’ (*l?aa dooc samput knoŋ pnɑt*) was prepared to grow into a woman of virtue to be chosen by a man who deserved to have her and to enjoy a harmonious marriage.

#### Cauls and Cords

Several features of early childhood, particularly in boys, were markers of a developing character of insouciance that could progress to violence. The same markers for a girl might signify that she would invite violence. A boy born wrapped at birth in the chorio-amniotic membranes known as a caul, or with the umbilical cord draped diagonally across his chest, might develop the feeling of powers of impregnability if their parents made offerings to their spiritual ‘master-teacher of the caul cord’ (*kruu sɑmnom sɑŋvaa*). But they would fall into a state of narcissistic omnipotence if their parents failed to do so, or if, as adults, they discontinued the offerings.

Monks offered remedies to fix the violated connections of the abusive men to their master-teachers, as if reflecting the abusive relationships with their wives.

Rith consulted a renowned monk-diviner who, to determine the identity of the master-teacher who had been violated, performed an augury ritual (*bool tien*). The augury pointed to ‘to the wronged master-teacher of the caul-umbilical cord’, which reminded him that he had been born in a caul, the umbilical cord draped over his chest. The monk prescribed that Rith urgently prepare an altar and make offerings to his master-teacher.

In fixing that broken relationship, the healer prescribed a substitution ritual. He had to place an effigy of Rith’s embryo made from items that resembled the umbilical cord or caul. The healer said that the umbilical cord should be draped from the rear to the front. Rith returned home and built the altar as instructed, placing the effigy in the centre of his platform. He hoped that he could get off the road to ruin. Within a couple of weeks, Rith felt better and he cut back on his abuse of women.

This case shows how some abusers see their abusive treatment of women as stemming from broken spiritual relationships with their master-teachers and that in repairing that relationship others are also repaired.

#### Birthmarks

A further harbinger of violence thought to be imprinted in the child was the mole. The omnipotence of men with birthmarks on the tongue or the penis, where they were known as *prɑcruy* of the Lingam, manifested itself when they ruthlessly trapped women like elephant hunters ‘lassoing their prey’ (*boʔrɑh cvak srəy*). The women were to blame.

Rith, a travel agent in Siem Reap, who abused women without fear of arrest, said his skin was invulnerable to the fangs of a dog or the venom of a cobra and immune to rabies. He said he had predictive powers and developed goose bumps as omens of inauspicious events such as police coming to arrest him for abusing his partner.

Some men who abused their wives explained their actions as a failure to fulfil their spiritual obligations to their former master-teachers.

Vibol, a car mechanic in Phnom Penh, went through a bad patch. He felt moody and made a mess at work. His wife taunted him, and he tried to strangle her and threw her into oncoming traffic on a busy road. Trying to understand why, Vibol recounted how he had been born with his umbilical cord draped across his chest – just like his father. Vibol’s parents were too poor to continue the offerings and settled with the homeless along the train line in Phnom Penh. Vibol said that his master-teacher had responded by placing ‘obstacles’ in his way, making him prone to bouts of anger, for example, drinking too much and fighting with his wife and in-laws, until he became ‘morally blind’.

In another way, husbands, and even some wives, believed that ‘women of misfortune’ (*srəy cɑŋray*) had a hidden mole somewhere, a ‘shy’ birthmark, perhaps on the vulva, which meant that these women, lacking in virtue, had ‘asked for it’.

Meanvy, a garment worker in Kandal province, never had any trouble in attracting men, but the courtships never lasted, ‘the fish always slipping off the bait’ (*rɔboot*). Her worried mother found a suitor, but the astrologer warned it was not an auspicious match because the groom was born in the Year of the Horse, ‘which is good at galloping’, and the bride was born in the Year of the Pig and would not be able to keep up with him. The parents were so desperate that they went ahead. Meanvy refused to let him consummate the marriage, until he complained to her mother who made her daughter agree to have sex. He became a drunkard, gambled away her parents’ money and savagely beat Meanvy until he was kicked out by her mother.

Meanvy’s second marriage broke down within 3 days. She clearly suffered ‘unlucky love’ (*ʔaʔpʰoap snae*), and could never keep a husband.

Meanvy needed a third husband to take care of her and her two children, but knew she would first have to cut the ‘unlucky love’. She consulted an astrological practitioner who diagnosed that it was the mole on her genitals that made her so unlucky in love and said she needed a substitution ritual to magically remove it. She would have to find a healer to scrape lightly on it with an incense stick and, taking a silver coin to represent the mole, hurl it away from Meanvy.

So Meanvy consulted Kiri, nicknamed ‘the healer that cuts moles’ (*kruu kat prɑcruy*) and ‘an elephant tamer love charm healer’ (*kruu snae maa dɑmrəy*) because he overpowered moles just like an elephant tamer charmed the elephant. He entered a trance and told Meanvy that his master-teacher had possessed him to tell him what to do. He probed her vulva, all the while reciting Pali stanzas. He said he would have to remove it in a dangerous ritual called *piʔtʰii kat prɑcruy*. Casting an ‘elephant tamer love charm’ on her vulva, he would collect their combined sexual secretions in a small plastic bag, which he would place on the altar to his master-teacher to activate the full effect of the ritual cutting of her mole. Desperate, Meanvy agreed. She removed her clothing and he removed his. He gave her the bag to hold and put quicklime and betel paste on her mole.

Meanwhile, Meanvy’s mother, Channary, came upon them, her daughter naked and the healer lying on top of her. She pulled him off and called the police. Kiri’s wife, Mealea, begged Channary to forgive him. Kiri stood trial and was imprisoned. Mealea borrowed $5,000 and bribed the police to get him out of prison, but nobody sought his services any more, and he abandoned his family, leaving Mealea with no way to repay the loan.

The search by Meanvy to explain the misfortune of losing two husbands led to the more profound tragedy of being raped by the person entrusted with the responsibility of treating her.

This sexual violence is exacerbated by the famous healer’s abrogation of his position of trust. It is complicated by his claim that he was simply following his former master-teacher’s instructions, a claim which, evidently, cut no ice with the court. Kiri’s crime spills over with adverse consequences for Mealea who, in standing by her man, has fallen into insoluble debt, and faces the opprobrium of her community and, ironically, abandonment as a fate shared with Meanvy.

### Times of Risk and Vulnerability-*Graha* and *Rasi*

Those involved in intimate partner violence viewed *krʊəh*, the Khmer concept of mishap or misfortune, as a motif that explained why they succumbed at certain times. Misfortune was more likely when a woman’s horoscope predicted an astrological zodiac house on the descent (*riesəy*). Women feeling that trouble was brewing would visit monks or healers who consulted manuals to clarify the category of the *krʊəh* and to prescribe ritual interventions to forestall misfortune.

Some wives thought that they were more at risk of violence at certain horoscopic times such as her ‘Zero Year’ (*kʊət cnam*), a period of one year during which the woman was vulnerable to disastrous events. Reasmey had heard that, to ‘cut the misfortune that had seized them’ (*kat krʊəh*), the best solution was to distance herself from her husband. She said leaving for a year saved the family from breaking up forever. Reasmey took her two children and left home to live nearby with her brother. During that year, her husband continued to urge her to return, but she held on until the year was up.

Some wives who had been abused would seek rituals to liberate the couple from their misfortune and stop their violence by cutting (*kat*), liberating (*rumdɑh*) or expelling (*bɑndəɲ*) the *krʊəh*. This was usually done in conjunction with the ritual to raise the *riesəy* by the pouring of lustral water over the couple, who would come back for top-ups on special occasions such as the New Year. Couples reported that they believed these rituals averted the violence, concluding that the violence could be stopped by a combination of civil human rights law (*ʔaanaacak*) and Buddhist doctrine (*puttʰeaʔcak*).

The village heads and case workers also reported cases in which fighting couples had reconciled, as conveyed in the couplet (*sɑmroh sɑmruəl*)—in accord and of one mind (*sɑmroh*), and easy and comfortable (*sɑmruəl*)—and who had signed an undertaking to stop the violence, until eventually the *riesəy* fell again.

### Abuser–Abused Incompatibility-*kuu kam*

Parents paid heed to the astrological compatibility of prospective unions. Young love-struck couples who fell into an ill-destined relationship (*kuu kam*, literally, ‘bad karma of the couple’), and did not care about their astrological compatibility, faced more than their fair share of misfortune (*krʊəh*), which could escalate into domestic violence.

In an ‘ogre family lineage’ (*pʊəŋ yeak*), a ‘wooden’ woman married to a man with a ‘fiery’ character would be consumed by his fire. ‘Fire’ could not marry ‘fire’ because the two would incinerate one another through mutual irritation and violence. In contrast, ‘water’ and ‘water’ were a fine match and did not lead to domestic violence. It did not matter if the couple were not deeply in love to begin with because, as people told us, ‘Harmony will grow into love’. As we learned from local people, ‘Love does not win over harmonious intimacy’ (*snae mɨn cneah snǝt*).

Young couples ignoring warnings of incompatibility did so at their peril. Parents of ‘modern’ daughters depicted the changes from tradition by inverting the old saying, ‘the cake is not bigger than the container’, to ‘the cake has become bigger than the container’. Mindful of the Khmer saying, ‘Uncooked rice once cooked can never return to its raw state’ (*ʔɑŋkɑɑ klaay cie baay tɨv haǝy*), mothers now realised these daughters were ‘spoiled goods’ whom no man would marry, and so capitulated to small ‘quasi-marriage’ ceremonies. These ill-matched couples were considered to have an increased risk of experiencing hardship and misfortune, and possibly violence, in their relationships.

Healers tried to forestall the violence by performing a substitution ritual in which the couple’s misfortune was transferred to an effigy, thereby freeing them to move on harmoniously together. Some monks also performed substitution rituals to release the misfortune (*rumdɑh krʊəh*). Interventions aimed to make the couple cool (*trɑceak*) and able to live together in harmony, ‘hand in glove’ (*trəv rəv knie lʔɑɑ*)—a bit like having good soil, good fertiliser, and a compatible plant in the soil (*trɑcum* means ‘to be fresh and cool’, or figuratively, ‘to be prosperous’). Village heads told us that sometimes these interventions reduced the violence.

### Violence that was Fuelled by Lust or Greed, Anger and Ignorance-*lobha, dosa, mohā*

Excess of the Buddhist ‘Triple Poison’ was thought to lead to intimate partner violence. The monks elaborated on sexual violence as fuelled by craving and greed (*rāga* and *lobha*), physical violence by anger and aversion (*dosa*), and violence generally by moral stupidity and delusion (*mohā*). The Buddha taught that the three fires of greed, hatred and delusion burn within us.

*Doṣa* was the most prominent poison fuelling violence. Fire, poison, pus and volcanic eruption were some of the metaphors used to describe the rage which, like oil in a frying pan, erupted when the flame was high and subsided when it was lowered. Some abusers felt ‘enraged to the point of vomiting blood’, as if they were wild animals devouring flesh. Some remorseful men realised their anger should not have been taken out on their wife, saying in Khmer, ‘Don’t melt lead in an earthen pot, only in an iron one’, suggesting that, if you pour your molten lava on your wife, you will ‘liquefy’ the marriage. Women, incensed by the violence, were eventually driven to murderous rage, as if that rage were a pustule about to burst. The gradient of intensity of the anger ran from ‘red’ to ‘black’ and, in a bodily gradient, from ‘blood’ to ‘liver’.

Alcohol fuelled fury (*kreev kraot*), entering the man (*sraa cool*) and taking control of him, as in the expression ‘*trəv sraa*’. He became ‘hot and irritated’ (*kdav*, *hǝl*), savage (*kaac*), not amenable to criticism (*niʔyiey mɨn baan tʰaa mɨn baan*, literally, ‘can’t speak, can’t tell’), *pah kʰəŋ pah kʰəŋ* (literally, ‘to hit or encounter anger’) and destructive and warlike (*kʰəŋ crɑlaot*). It burned like fire inside (*kAmraol*, derived from the root *roul,* which means ‘barbeque’), and he behaved as if possessed and made berserk by a ferocious spirit (*kAmraol cool*). Some said he had a blue–green face, a sign that the toxin in the blood had damaged the gall-bladder. He became *prɔhəən kaoŋ kaac*, initiating foolhardy acts (*prɔhəən*, literally ‘over-reaching one’s limits’), and continued with *kaŋ*, literally being ‘warped’ and disdainful, until finally he became violent (*kaac*). The enraged man who beat his wife behaved as if infused with an evil spirit, or *kɑmraol*, and the healer, in a ritual intervention known as *baoh kɑmraol*, might ‘sweep out [as with a broom] and destroy by fire (*baoh*) the evil spirit (*kɑmraol*)’ and try to bring the abuse under control.

*Mohā*, ignorance and delusion, is the third poison and, in Buddhist teachings, the abuser’s mind becomes deluded through ignorance (*avijjā*), causing perversions that take what is painful (*dukkha*) as pleasurable. That said, beating a wife would not trouble the perpetrator in a state of *mohā*. The gradient of impunity, of at the least, freedom from remorse or guilt, is shown here in body metaphors, such as men having ‘no heart’ or, worse, ‘no liver’ or, at the very worst, ‘no heart no liver’.

### The Road to Ruin-*apāyamuk*

The downward spiral into violence was called ‘entering the road to ruin’ (*apāyamuk*) and often involved alcohol abuse, which was central, although closely followed by the increasingly damaging use of illicit drugs and pornography, womanising, gambling, corruption and consorting with gangs and people of ill-repute.

Demoralised out-of-work men, smarting from their loss of status and ruefully observing their women becoming the breadwinners in garment factories, became resentful and driven to drink. The spiral was accelerated by pawning the wife’s property without permission or by borrowing money from their wives and returning only a part and beating them when asked to do so. Men got blind drunk to alleviate their anger (*pʰək sraa rumsaay kɑmhəŋ*) and to rid themselves of their suffering (*pʰək sraa bɑmbat tuk*) and became fearless and cocky (*prɔhəən*), expressed as ‘big heart’, ‘big liver’, or having a ‘black heart-mind’ and ‘black liver’.

After a drinking session, Sothiya couldn’t be bothered going to work and supporting his family. His wife, Chanlina, said his anger was like a coiled spring (*kʰəŋ cralaot*). Enraged (*tup lɛɛŋ baan*), Sothiya rushed at Chanlina and beat her up. He was, in Chanlina’s words, ‘*crɑlaot cʰaŋ*’, his aggressive behaviour suggestive of the agitated clashing of cymbals. Chanlina became ‘*kʰəŋ crɑlaot*’ too, rushing in to counterattack, saying, ‘The cymbals are clashing, the cymbals are clashing,’ (*crɑlaot cʰaŋ crɑlaot cʰaŋ*). She loathed her beastly husband, attacking Sothiya, saying, ‘You low animal, die!’ (*?aa ŋoap*). No one intervened.

The wives of these drunken men described them as inflammable. Some believed that sexual violence was brought on by watching pornographic videos. Men who disrespected the territory of the guardian spirits risked retaliation by the offended spirit possessing him (*kɑmraol cool*) and driving him to abuse his wife. When these men flared up with rage (*niʔyiey kʰəŋ, niʔyiey kʰəŋ*), simply initiating a conversation would infuriate them (*pah kʰəŋ pah kʰəŋ*). One man, for example, addressed his wife like this, ‘You, evil slut! (*hoŋ* … *ŋaeŋ*, the rudest form of address), you did this, you did that!’ (*ʔiceh ŋaeŋ ʔicoh*, *slang for ʔɑɲceh ʔɑɲcoh?*), then threw household objects around in such a fury (*baok prah*) that everything was smashed to pieces (*baok prah ktɨc ktoam*). Sooner or later, they would beat their wives and any of their children who got in the way. The men blamed their *apāyamuk* for robbing them of insight and judgement.

We turn to the story of Chavy, the woman born in a caul with the umbilical cord across her chest.

Chavy met Yaem, a monk of 15 years and an accomplished teacher of *tripitaka*. Surely, she thought, here was a ‘five *hat*-chested man’, a good catch. Interested in her, Yaem left the monkhood.

Chavy’s mother was keen on this marriage. Yaem had learned magic such as how to cast a love charm to ‘soften the heart’ (*bɑntʊən cət*) of Chavy and her mother. They married and lived in harmony, doing good works for the temple. Yaem was a supportive husband. Once it was time for babies, he took up work as an electrical repairman and started visiting clients away from home.

All went well until the altar was placed disrespectfully near the marital bed. At once, Chavy’s master-teacher withdrew his protection, and she became vulnerable to disaster. Yaem ignored her warnings and, instead, took up heavy drinking and entered the ‘road to ruin’. Chavy started to remember the gossip about the kind of person he had been before she had met him, quoting sayings like, ‘If your long shirt had been soiled by mud and you didn’t remove it, you would stink of piss and shit (*klən c?eh c?aap*)’.

The couple were family friends with Molika and her husband Boran. Molika was the sort of woman who attracted married men. Boran could not control her and seemed to allow her to beat him regularly. Though older and far from beautiful, she ensnared married men by putting love philtres (potions) in their food.

She told us how Molika had ensnared Yaem. She used syrupy words of seduction, as in the expression ‘*niʔyiey pʔaem lhaem cak ceɛk cak skɑɑ*’ (literally, made with ingredients of banana and sugar). She got a love charm from a healer which included her menstrual blood, and Yaem developed ‘madness of love sickness’ (*ckuət* *snae*). He seemed to have been driven berserk by a spirit, developing ‘madness of confusion’ (*ckuət* *vʊəŋveiŋ*) and clouded judgement (*mohā*). He abandoned his home life to spend time having endless sex with Molika.

Now, goaded by Molika, Yaem tried repeatedly to kill Chavy. He would kick her in the ribs, stab her in the belly, pound her on the chest and club her head to the point that she regularly vomited blood clots. Molika eagerly joined in the beating and urged Yaem to burn Chavy alive.

In despair, Chavy beseeched him not to drown in ‘unwholesomeness’ (*kilesa*) and the ‘Triple Poison’. She begged him to have pity on her, on his children, and even on Boran. She said, ‘You are my husband and you cannot be with another man’s wife, and someone else’s husband cannot be mine’ (*pdəy yəəŋ prɑpʊən kee pdəy kee prɑpʊən yəəŋ*). She told him that he would end up in hell (*kmien kɑmnaət*, literally, ‘cannot have a birth’), but her entreaties only inflamed him more.

Molika hired a sorcerer to cast a spell to kill Chavy. She developed excruciating pain all over her body and saw huge insects, including butterflies the size of a hand, penetrating her skirt to viciously attack her genitals. At night she saw a witch, or *?aap*, an ugly figure of a disembodied female with messy long hair and a bunch of her intestines and her liver trailing from her neck.

Chavy hated Molika, ‘that low-down bitch woman’ (*mii srǝy nuh*) with ‘a black face’. She became suicidal and doctors could not help. A traditional healer gave her some relief.

Still wanting to keep the family together for the sake of the children, she ran to the local authorities asking them to counsel (*ʔɑp rum*) her husband, but Molika had already bribed them, and they warned Chavy to back off or she might face a defamation lawsuit. Even the cuckolded Boran accused Chavy of slandering his wife and claimed compensation of one million riels. Chavy was outraged to the point of wanting to chop them into pieces and put a curse (*dak bɑndaasaa*) on them and, sure enough, several of them were subsequently killed in motorcar accidents.

Chavy wondered why she would have made such a bad choice. She remembered that during their courtship Yaem hardly ever opened his mouth wide and, looking back, she concluded that he must have had a Sarika love-charm implanted in one of his teeth which magically imbued him with seductively sweet and irresistible speech. She felt such a fool.

Finally, Chavy had to flee from home with her children and live on the streets, which she dubbed ‘Torture Fields’. She was taken in by a temple monk, where she got some relief from the ritual pouring of lustral water by the monks and realised that her life was similar to that of Pātacāra, the two of them driven almost to madness.

The road to ruin was smoothed by the actions of the lover who used every trick, including magic, to make the man lose his way and commit emotional and physical abuse on his wife, who was grievously wounded by his adultery (*saʔhaay smɑn*) and betrayal (*ʔɑmpǝǝ pʰət kbɑt*).

#### The Extended Family Embroiled

In day-to-day life it is inevitable that family members, like plates in a bamboo basket, will knock against one another and eventually become chipped and, while sometimes they may break, as in domestic violence, they do not invariably shatter, and the proverb seems to give voice to a cultural norm. This image is how the wife might accept the day-to-day disagreements and tensions (*rɔkam rɔkaoh*, literally, ‘irritated and grated’).

In order to make ends meet, Samphy resorted to work at the M &V garment factory far away in Kampong Chhnang province. Phirum lost his job and had to stay at home, where Samphy expected him to do household chores, but he became angry when she insisted that they ‘help each other with a hand or a foot’ (*cuəy knie muəy dey muəy cəəŋ*). He said her task was to clean the house and he refused to join the meal table saying, ‘Our hands and feet are [no longer] joined up (*day cəəŋ mɨn coap knie*)’ and he started beating her.

Resentful men like Phirum, caught between the demands of wife and mother-in-law, felt ‘anger that chops off the head and the tail’ (*kʰəŋ dac kbaal dac kɑntuy*), like a decapitated lizard thrashing around blindly, and the relationship with his wife became *rɔkam rɔkaoh*.

The more plates in the basket, the greater the likelihood of the crockery colliding. In an extended family, the basket is loaded with husband, wife, mother, mother-in-law and others. The radius of violence could even include the ancestral spirits on both sides. The conflict could be ignited by the man offending his ancestral spirits, the conflict spilling over between the ancestral spirits of both parties. It was worse, as shown in the following case, when there was a racist element.

Vibol’s mother identified herself as looking pure Khmer, with dark skin. She said her father was a proud fighter against the French colonialists, and even her grandfather was a dark, pure Khmer who looked like a figure of ancient Angkor. Vibol’s wife Sokha was part Chinese–Vietnamese. The proud mother never accepted the lower-grade wife, and they fought constantly.

The conflict spread. Sokha’s mother blamed Vibol’s parents. She felt that Vibol’s mother arrogantly saw herself as from a ‘top drawer family’ and viewed their family as impoverished scum dependent on remittances from their relatives in North America. Vibol felt caught between wife and mother and, to escape, would set off for drinking session with his mates.

When Vibol threw Sokha into the path of oncoming traffic, his parents-in-law instantly ran out after her, ‘swarming like bees reacting to an attack on their hive’. Vibol puffed out his chest like a wild cock and assailed his father-in-law who replied, ‘Do you want to kill me too? Or do you want to treat me as a father? You attacked my daughter, what else do you want to do to me?’ and threatened Vibol that he could never take his daughter back as his wife.

Sokha’s parents saw that Vibol’s mother did not lift a finger to restrain her son from killing their daughter, and they poured scorn (*prɑmaat*) on her, saying, ‘You, you black cunt (*mii kɑnduəy kmav*)!’ alluding rudely to the Khmer blackness of her genitals.

Now Sokha’s parents could not stop themselves from turning on their son-in-law Vibol, ‘Go fuck your mother, the bitch! (*ʔaa coy mraay* [*mdaay*]!) If you dare come back to our daughter, you’re a dog.’ They portrayed mother and son as a pair of shameless *tiracchāna*, animals who walk on four legs rather than upright. He told us it was they, not his mother, who had black genitals, and they put a curse (*dak tumniey*) on her. Relations with his in-laws seemed mortally severed. Sokha’s parents resolved that she had to leave him that night, and she did, taking her younger son with her. They reported the attempted murder, but they could not afford the going rate to bribe the police.

#### Help-Seeking

Love charms played a role in efforts to end the violence or, at least, restore harmony.

Samphy decided to leave her abusive husband, Phirum. His mother was so upset she asked a medium to cast a love charm to reconcile the couple. The medium recited the *gātha*, ‘Come here, I invite you, the Deity that Binds, the Deity that Keeps the Binds Bound’, and then inserted the name of her daughter-in-law, Samphy. She finished with words in Pali, ‘Come here, *sārikā* Lin Thong’, a standard image for a love charm and an allusion to the mythical blackbird’s enchanting song. Then she prepared twelve copies of a ‘love charm’, *Āthabbaṇa yantra*, which were metal squares inscribed with male and female letter pairs that were rolled along a magical hip cord and which she gave to Phirum’ mother to take home for him to wear.

Interventions such as these could change the momentum, helping the wife to feel more optimistic and changing the dynamics between the couple. In Samphy’s case, she returned home and resumed normal family life, and her husband stopped his abuse. She stayed at home for a year until eventually the violence returned.

Vibol consulted a medium named Davi, who cast a love charm to imbue the ancestors on both sides of the family and their descendants with loving-kindness (*metta*) and empathy (*ʔaanət*). The ritual ran at midnight for three successive nights. She called all the souls, including the ancestral spirits, who were caught up in conflict with Vibol. She recited the *gātha*: ‘Om, Great Nostalgia’, to empower Vibol’s face to call his enemies to him and turn their hatred into love and friendship. Davi prepared a *yantra* known as ‘*yantra* to splint and knit the fractured ancestral spirits’ (*lumʔɑɑp cɑmbuə*). Davi had meanwhile prepared a ritual hip girdle for Vibol which was awaiting him in a bowl of clear water and which would ‘cool off’ any residual anger in Vibol’s mind as well as in those, such as his parents-in-law, who were antagonistic towards him. She performed a ritual pouring of lustral water on Vibol and his wife and children. Within a week, harmony was restored between husband and wife, the in-laws and the two sets of ancestral spirits.

On follow up, perpetrators acknowledged that their violent behaviour had been caused by the ‘dark road’. Now that they had completed the treatment, their astrological fortunes (*rasi*) remained elevated, their economic position improved and the cycle of violence was broken. The pouring of lustral water had cleansed their minds of their earlier rage.

### Mental Aberration-*Mohā*

*Apāyamuk* can flow into the ‘cultural attractor’ called *mohā*, a loss of insight and judgement leading to a deranged mind. The agitated man’s deranged thoughts become dark and dirty (*ʔuə ʔap*), and his confusion mounts (*kvɑl kvaay*). He grows more resentful (*muə maaŋ*, literally, ‘dark and impure’), the smouldering state of mind (*muə mav*, literally ‘completely dark’) now like tinder and, bursting into flame (*mue mav kdav krahaay*), spilling over violently onto the wife and children (*daal vaal*, literally ‘spreading like wildfire’).

Some perpetrators experienced this state pounding inside the trunk like a leaping fresh-water fish, or as sitting in the chest and falling with a thud to the stomach, ‘the heart sinking to the liver’. Some survivors depicted their emotional pain as if their body organs were plugged, their painful feelings bottled up. There was a gradient of intensity from ‘corked blood’ to a more intense suffering called ‘corked’ or ‘black’ liver or ‘liver pain’.

### Violence Without Guilt or Remorse-Moral Blindness-*Mo baŋ*

The violence escalated with the development of moral blindness, *mo baŋ*, the eighth cultural attractor. The men abused women with impunity, thick-skinned (*muk kraah*, literally, ‘thick face’) and with faces impervious to feeling compassion for their womenfolk (*muk klaɲ*, literally, ‘face covered in lard’). People depicted *mo baŋ* in popular expressions as, ‘The monk donned the saffron robe but failed to shave his scalp. The layman looked in the mirror with his eyes closed’ (*sliek sbɑŋ mɨn kao sɑk cloh kɑɲcɑk tmɨc netrar*). They hid behind *mo baŋ* as a way to diminish their accountability.

Victims of abuse could also suffer *mo baŋ*. In a popular song (Moun Mara 2012), the crooner sings of a cuckolded man, ‘Why had I become such a person with eyes that did not see as if they had no pupils? I did not see my sweetheart’s copied heart.’ She had cast a love-charm to make him *mo baŋ*. It was so bad that he jumbled fragments of the *dhamma*, singing, ‘Dhammaŋ, sanghaŋ, buddhoo buddhaŋ’ instead of the correct sequence, ‘Buddhaŋ, dhammaŋ sanghaŋ.’ Now he realised that he was *mo baŋ*. Stricken by shame, he wanted to kill himself.

The monks, ritual officiants, Buddhist devotees and anyone else who studied the *dhamma* viewed moral blindness as a breakdown in *hiri ottappa*, or loss of conscience.

It was sometimes difficult to differentiate regret and remorse. An abuser might say he was *saok sdaay*, literally, ‘lamenting’ and felt ‘regret and sorry’, ‘*kmaah kee*’, to mean they felt ashamed or embarrassed in front of others, and *kmaah kluən ʔaeŋ* when they knew themselves that what they did was wrong.

One night, Rith had a dream in which he saw himself picking up some stinking fermented fish-paste and later trying to wash the smell away. He put more fermented fish-paste on his hands, but his efforts were in vain. Then he dreamed that he had belatedly taken a food offering to a monk in a temple. When he woke, he interpreted his dream as meaning he could not rid himself of his ‘bad smell’ except by taking the road to the monkhood. Rith reflected on the cycle into which he had fallen. He had led a clean life after disrobing from the monkhood but eventually he had entered the road to ruin once more.

Such men were tortured by their inability to wash away the blame. Remorse (*rʊəŋkiəh cet*) on its own could not end the violence.

## Discussion

The popular cultural perceptions of violence against women, in Khmer *ʔɑmpəə həŋsaa ləə strəy*), are deeply embedded for both men and women in Cambodia. To speak of GBV in Cambodia, it is essential as a starting point to be clear what Cambodians and westerners mean by the term ‘gender’, which the Ministry of Women’s Affairs (2014) defines to be the socially constructed attributes and opportunities associated with being male or female. Here is a challenge, for in Khmer the difference between male and female is expressed by the Khmer *pʰeit*, but there is no word for ‘gender’, and contemporary NGOs have had to adopt the loanword ‘*zenda*’ from the donor language English. As Aveling (2012) showed, ‘gender’ is viewed as a foreign word associated with international NGOSs; the concept remains relatively novel, with some elements being rejected as inapplicable to Khmer society and others hybridized with traditional knowledge.

In this section, I discuss the epigenesis of the violence, followed by some practical implications.

### Epigenesis of Violence Against Women

**Fig. 2:**
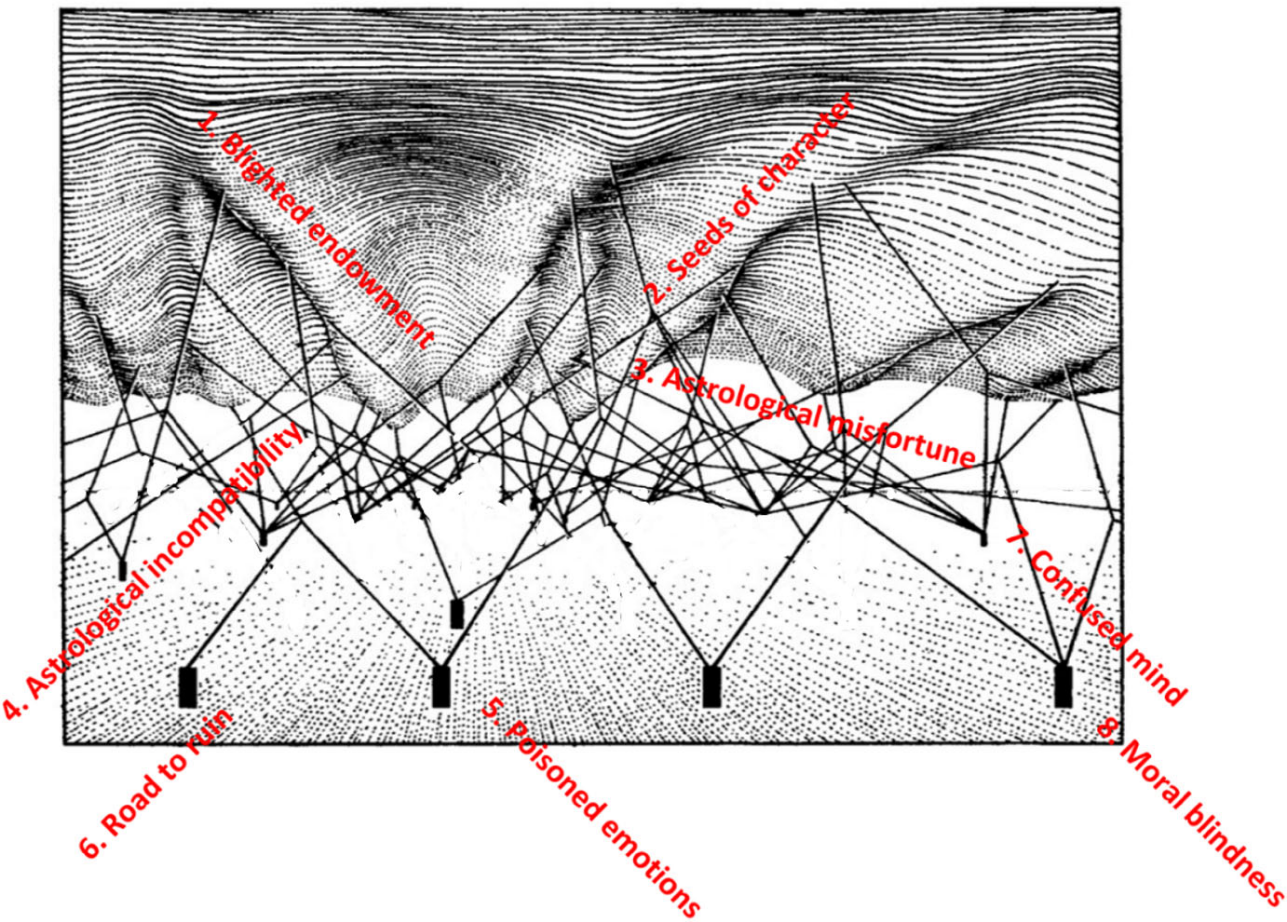
Complex system interactions underlying the epigenetic landscape, showing ‘cultural attractors’

The landscape of violence against women is underlain by a network of interacting elements and ‘the topological relations of the interacting pegs and guy ropes determine the architecture of the visible undulating landscape’ (Tavory, Jablonka and Ginsburg 2012:12). The pegs, such as blighted endowment or the road to ruin, are the ‘cultural attractors’ towards which the ball rolls, but they can move and interact according to contemporary social changes (Fig. 2).

Violence against women has form and structure. Its epigenesis is pulled into shape by the guy ropes and pegs that connect the cultural attractors and direct the course of the rivulets in the epigenetic landscape, one ‘cultural attractor’ after another. The rivulets could be considered to be the course of individual people’s lives, and they are also larger, the patterns of social and cultural practices. It starts with blighted endowment and continues as seeds of character, emerging as astrological forces. It erupts like poison and develops once the perpetrator enters the road to ruin, a powerful central ‘cultural attractor’. It sets the stage for the act of violence as the perpetrator’s mind becomes confused and seals his fate with the onset of moral blindness and an absence of shame and blame.

The story of Chavy shows a case in which all eight cultural attractors are represented in the epigenesis of her situation. The scripted violence in her previous life led to her destiny in encountering a seemingly perfect man, a ‘five *hat*- chested man’ who was such a morally inferior choice of husband. The second cultural attractor she acknowledges is her childhood marker of violence, first through the character of her husband and second through her special characteristics from birth and her former master-teacher whom she later violated. The third cultural attractor is the misfortune she experienced in choosing the immoral man over the virtuous suitor. The fourth cultural attractor she is drawn to is the mismatch in the marital couple, and she ruefully acknowledges all the warning signs such as the dream and the astrologers’ misinterpretation of the horoscope data. The fifth cultural attractor that gets in the way of a harmonious life is the ‘Triple Poison’, the lust of the husband and the craving of the temptress, the anger of everybody, and the delusion of the husband as he falls under the evil spell of love cast by the magician–sorcerer. The sixth cultural attractor is the ‘road to ruin’, prompted by a change in the social roles of the couple as they moved from a family of two who frequented the *wat* to a growing family that needed to make a living in the secular world, leading to exposure to alcohol and bad company. The seventh cultural attractor is the madness and confusion that resulted from the love charm, and the final cultural attractor is the ‘moral blindness’ when under the influence of evil spirits the husband became shameless and blameless.

#### Blighted Endowment

The popular Cambodian Buddhist view is that those who die a tragic death, especially those whose final moments are violent, will have difficulties making the transition and having a positive rebirth (Holt 2012). The results here show that committing violence from beyond the grave is a powerful driver for ‘identifying with the aggressor’ by turning the passive experience of being hurt into the act of hurting others. The case of Chann shows how people might explain the actions of a man who perpetrated violence against women as a replication of that which had surrounded his previous generation and shaped his father’s character and was transmitted to the next generation.

The millenarian ‘Buddha Predictions’, the *Buddh Damnāy* (de Bernon 1994), forecast endemic violence in Cambodia (Hansen and Ledgerwood 2005). People draw on these predictions as a way of steeling themselves against unbridled violence, including that against women, and their ideas are given expression in *yantra*, such as the ‘Three Vast Plains of War, Famine and Disease’ which were drawn by monks to help dispirited survivors (Eisenbruch et al. 2013). Rather than simply befalling individuals, violence can affect large numbers of people or even the nation as a whole.

#### Character

Character, or *caʔret*, from the Pāli *carita*, has three shades of meaning in Cambodia. It is rooted in a person’s predestiny in the previous life (*karma*), coloured by its genetically shaped breeding stock (*puuc*, from Sanskrit *bīja*), and further shaped by the way a child has been reared in a particular family milieu (*pʊəŋ*, literally, ‘its pedigree’, from Pali *vaṅsa*). Contrary to formal Buddhist doctrine, character is also believed to be shaped by the position of the planets and the stars and the child’s year of birth.

The caul or the umbilical cord is taken seriously in traditional societies. In Malaysia, a child born with the cord around the neck had to be ritually released from the dangers inherent in its hazardous entry into the world (Laderman 1987). In Cambodia, parents of such a child were traditionally expected to follow codes of conduct such as making offerings to the child’s spiritual master-teacher known as a *kruu baatyiey* ​(Sanskrit *upādhyāya*) or to a magical master-teacher, the *kruu rɔbiən* (Eisenbruch 1992).

A further example of a harbinger of violence thought to be imprinted on the child is the mole. In Buddhist societies, a mole, far from being trivial, was regarded as predictive of the child’s nature in adult life, often a grave portent of character and a marker of violence (Jungwiwattanaporn 2006; Flint 2011).

#### Astrological Misfortune

*Krʊəh* is a concept deeply embedded in the Khmer psyche. It had to do with having been seized by a particular constellation of the nine heavenly bodies and known as *nup krʊəh*. The demon seizes its hold upon the sun or moon—or upon a human ‘seized’ for better or worse by their astrological destiny. Reynolds (2016) describes the sciences of prognostication in Buddhism, such as astrology, and the interpretation of birthmarks, as ‘deployed to help people face up to unpredictability in life’.

*Krʊəh* is a foundation of fateful problems, including violence against women, and used in expressions such as *nup krʊəh* (nine predictions of misfortune), *krʊəh cɑŋray* (misfortune) and *krʊəh kaac* (savage misfortune). The results show how people seek to curb astrologically shaped violence in the context of a combination of civil human rights law (*ʔaanaacak*) and Buddhist doctrine (*puttʰeaʔcak*). Work (2014) found that villagers trust monastic authority and integrity more than democracy and that ‘*ʔaanaacak* can’t control the fighting, but *puttʰeaʔcak* can … They are different. The Buddha controls the heart; the police control only the body’.

#### Astrological Incompatibility

Some marriages are ‘meant to be’ and are known as ‘*kuu preeŋ​​​*’. The couple is destined to stay together ‘until they turned 100’ (*ʔaayuʔ mrɔɔy*), as reflected in songs popularised by the singer Sin Samuth. The Jātaka legend of Vessantara and his wife Maddi who stayed strong (Penner 2009) similarly conveys the idea of a marriage made in heaven.

Inauspicious unions, however, are *kuu kam*—prone to misfortune and violence in the course of the marriage. The prototype of such a marriage is the legendary Jujaka and Amittada who, unlike Vessantara and Maddi, were thrown together inauspiciously when Amittada’s father, because of debt, offered his daughter to another couple. As a result, she endured a marriage filled with conflict and violence.

An abuser can project the blame on his wife. The best match, to a bride classified as completely virtuous (*srəy krup leakkʰaʔnaʔ*), would not lead to violence: ‘In comportment, she would be soft and sweet; in activities, she would obey and serve her husband as she had been to her parents, and in sexuality, she would be faithful to her husband’ (Amratisha 2006). The alternative would be a woman whose character was not completely virtuous (*srəy kʰaat leakkʰaʔnaʔ*), who would damage the groom’s good fortune (*kʰaat liep*) and make him go down the road to ruin (*apāyamuk*) or, as some said, to defeat and destruction (*paʔraa cey*), a Khmer term that reflects an archaic concept, *parā*, in Sanskrit.

As for the character of the husband, he could bolster his character through becoming a monk for a short time, learn Buddhist morals and qualify as a ‘scholar’ (*pandita*). When he left the monkhood, he would be called a completely virtuous man (*proh krup leakkʰaʔnaʔ*) and suitable to be an exemplary husband. The harmonious marriage would be one in which the husband was *proh krup leakkʰaʔnaʔ* and the wife a *srəy krup leakkʰaʔnaʔ*. A qualitative exploration by GADC (2010) of gender norms, masculinity and domestic violence is entitled ‘The five *hat* chest’. *Hat* referred to the 2.5 m. length of cloth that a monk wears across his chest. Married men depicted the ideal or strong man as *proh daəm truuŋ pram hat*, literally meaning the five-*hat*-chested man, whereas an incomplete man was only *pii hat*, or a two-*hat*-chested man. The man who had a ‘five *hat* chest’ was one with the ideal hegemonic masculinity, knowledgeable in the *dhamma* and of exemplary behaviour.

In each case, the role of the astrologer in predicting and explaining disharmony is taken seriously. The marriage books, such as the Vibhīṣaṇa manual of astrology (Haoraayu 1973), are still sold in markets. Reynolds (2016) depicts Buddhist divination as ‘a semiotic science of reading signs that is not so much about predicting the future as about making decisions that lead to favourable outcomes’. But astrology, no more than any other cultural attractor, should not be misappropriated to paralyse a couple. As for an abusive man, as pointed out in Buddhist family counselling (Shaneman 2008), he must take responsibility for his actions just as his partner must for remaining immobile in the face of abuse.

Neoliberal modernity plays a part in weakening the traditions of betrothal. The sentiments of the mothers disappointed with their daughters’ break with tradition are echoed in the seventeenth-century story of the beautiful maiden, Teav, and her lover, the young monk Tum (Chigas et al. 2005). Mothers in Cambodia would traditionally warn their daughters that ‘The cake is [should be] no bigger than the *niel*’, the coconut shell used as a mould to measure 600 grams of rice, meaning that a daughter should not break with tradition by following her heart because that would court a *kuu kam* marriage which could lead to violence. But in today’s scenarios, where the daughters shrug off tradition by hooking up with whomever they choose, the mothers say, ‘The cake has become bigger than the *niel*’, and the consequent astrological mismatch between a husband and wife is viewed as an explanation for domestic violence.

#### Triple Poison

It is clear from Buddhist teaching how craving spills into anger and how each is a forerunner of violence. The Triple Poison, in Khmer the *tnam pul bəy yaaŋ*, are the three unwholesome roots, in Pāli the *akusala*-*mūla*, the toxins of the mind. The Buddha taught that the three fires of greed, hatred and delusion burn within us (Wayman 1957) and push people to mental aberrations that could lead to abuse. The Buddha said, ‘Holding onto anger is like drinking poison and expecting the other person to die’. Legends paint the choleric person as a wild tiger, snake or demon, even a *tiracchāna*. The Buddhist post-canonical Ghatva *sutta* discourse, ‘Having Killed’, associates the poison with ‘the sweet tip of anger’ which drives people to hurt others, even their loved ones. The poison lodges in the abuser’s heart and re-inflames him (Eisenbruch 2010). As Chuon Nath wrote, ‘Anger is wrong, anger spoils, anger makes you suffer loss and ruin’ (*kʰəŋ kʰoh, kʰəŋ kʰooc, kʰəŋ kʰaat*) (Chuon Nath 1967). The ghatva *sutta* provides the recipe for cure, which rests on the slaying of anger itself. Nowadays, the concept of Triple Poison has even influenced activists in promoting peace in western settings (Madden 2010).

We see how the emotional abuse of women is so strongly linked with betrayal, just as the Buddhist legends clearly show the links between poison and betrayal (Davids 1989). Devadatta, pretending that he was full of good intentions, kept trying to poison the Buddha. This is the origin of the Khmer saying, ‘He has the heart of Devadatta [the ambitious deviant monk who fomented violence], and the mouth of a Tevada [the peaceful celestial being]’ and is a popular way of depicting duplicity in marriage and politics. Another expression for seductive duplicity leading to violence is ‘words of the fig’ (*sɑmdəy plae lvie*), meaning that the fig looks beautiful on the outside, but inside is rotten, full of midges (*mɔmʊəŋ*).

*Mohā*, ignorance and delusion, is the third poison and, in Buddhist teachings, the abuser’s mind becomes deluded through ignorance (*avijjā*), causing perversions that take what is painful (*dukkha*) as pleasurable (Dhammasami et al. 2012). Beating a wife would not trouble the perpetrator in a state of *mohā*. Abusers are oblivious to the reciprocal relationships with their wives as victims.

#### The Road to Ruin

Entering the road to ruin (*apāyamuk*) is a powerful peg—possibly the central one—in the epigenesis of violence. Indulgence in ‘alcohol, women, and gambling’—paths likely to be taken by impoverished, unemployed and desperate men and women—invites self-destruction (Kitsripisarn et al. 2013). Buddhism has various taxonomies of *apāyamuk*, a popular one being ‘The Six Bad Habits’: being lazy, gambling, watching bad entertainment, going out late at night, consorting with people of ill-repute and addiction (Radich 2007). Of these, liquor features as the fifth of the Five Precepts to be observed by all Buddhists, ‘I undertake the training rule to abstain from intoxicants causing heedlessness’, and getting drunk is a sure way of becoming heedless to the effects of beating your wife. In more down-to-earth language, liquor in Khmer is called *ʔaakaa saahaav*, literally, ‘savage characteristic’, which colloquially is called *?aa kaac sahaav*, literally ‘vicious savagery’, and alcohol is the hallmark of entering the road to ruin, though on its own it does not directly cause violence. Contemporary research in Cambodia shows that a husband’s alcohol use is associated with both physical and emotional violence (Eng et al. 2010). Government rhetoric on the perceived causes of domestic violence tends to focus on alcohol and poverty. A raft of national-level surveys has tried to establish the basis of violence against women in Cambodia (Banta et al. 2012). Alcohol use is linked to coercive sex and domestic violence. In her work on gendered experiences of drunkenness and violence in Cambodia, Brickell makes plain the complexity in cause-and-effect between alcohol and abuse; she explores the ‘vocabularies of motives’ used to differentiate alcohol use and violence and sees the underlying inequality between men and women, rather than the alcohol itself, as causing gender-based violence (Brickell 2008).

Entering the road to ruin remains a powerful and popular image in Theravada Buddhist countries, where social media warn of its dire consequences. The *Parabhava sutta*, the Discourse on Downfall, explains *apāyamuk*’s twelve causes and how to avoid them (Premawardena and Premawardena 2010). Buddhist teachings on *apāyamuk* are seizing the popular imagination in Thailand (Parnwell and Seeger 2008). In Cambodia, warnings about *apāyamuk* were first published in 1947 (Master Venerable Seyrey Samphorn Kim Tho 1951) and they still apply today. Every year on All Souls’ Day, monks preach the Discourse on Downfall, and it is chanted daily on the radio during those 15 days.

An abuser down the road to ruin wrecks the life of his intimate partner. Cases of abused women such as Chavy take us to the story of Pātacāra, a well-known story of a woman who lived in the time of the Buddha who was driven mad by the loss of her family and is emblematic of the misery that can beset women. In cases of intimate partner violence, the loss of husbands arises from human disasters such as murder, or by being taken away by rival women, who are commonly likened to a bird of prey, such as the wild fowl (*moan prey*) or the kite (*klaeŋ*). Some of the women abandoned in this way end up, like Pātacāra, crazed.

#### Mohā

Whatever has happened before—greed, fear, anger, ignorance or entering the road to ruin by excessive alcohol consumption—the person becomes mentally confused, the mind in darkness, a state called *mohā*, from the Sanskrit and the Vedic word *mogha*, meaning ‘empty’ (Rhys Davids and Stede 1900).

#### Moral Blindness

*Mo baŋ* means complete darkness, thoughts mixed and muddled, the man not knowing right from wrong, the ‘empty mind’ (*mohā*) becoming shut off (*baŋ*) from awareness (Chuon Nath 1967). An important aspect of moral blindness is the way in which, with time, people develop a shifting pattern of ‘social memory, violence, trauma and morality’ (Kent 2015). The media picks up the theme of moral blindness. As mentioned earlier, in a popular song (Moun Mara 2012), the crooner sings of a cuckolded man lamenting his inability to see the truth about his sweetheart who had cast a love-charm on him. Realising this, that he was now *mo baŋ*, a donkey, he felt stricken by shame and wanted to kill himself.

Moral blindness leads to feelings of impunity (*nitoandeaʔ pʰiep*), a word that finds its root in ancient usage, meaning ‘the position of no penalty’, literally, ‘no rod’. The Sanskrit *daṇḍa* was a bat or cane used by the king in ancient India as an instrument of punishment. Cambodians use the Khmer word ‘*touh*’, which is embedded in Indian mythology and Buddhism, and means a ‘pastiche of fault, blame and punishment’ (Headley, Chim and Soeum 1997).

*Hiri ottappa* is a compelling Buddhist explanation for the insouciance of a morally blind person (De Silva 1976). *Hiri* is a healthy sense of shame which deters a person from committing violence. *Ottappa* is the fear of blame or moral dread that deters a person from committing violence through fear of recrimination. They do not refer to guilt over past violence, but as bulwarks against committing it. While a person is morally blind, he is incapable of noticing *hiri ottappa*. Although few laymen were familiar with the formal terms, everyone could articulate the ideas of *hiri* and *ottappa* in their ordinary speech.

### Stigma

Stigma is among the most significant barriers to intervention for GBV (Bent-Goodley 2007), and it is essential that the local experience of it is grasped. Stigma as a cultural process is essentially a moral issue ‘in which stigmatized conditions threatened what really matters for sufferers’ (Yang et al. 2007:1528). This article shows the place of family secrecy and how and why violence against women in Cambodia is not supposed to be talked about. According to the Codes of the Woman, the three ‘Mounds of Fire’ (*pnʊək pləəŋ*), or bonfires, are the ‘three sources of contention for a married woman (family, husband, in-laws)’ and ‘Women should not bring “fire” (conflict) from outside into the house, or take fire inside the house outside, and they should take care not to spread or overheat fires’ (Derks 2008). *The Handbook for Village Facilitators* misinterprets this saying to mean simply that ‘a woman should be submissive to her husband and that domestic violence is an internal affair’ (Ministry of Justice and Ministry of Interior 2010), but the sense of the saying is more subtle than that. In the end, stigma and the cultural imperative to keep family problems out of view by ‘keeping quiet and doing nothing’ to preserve family harmony (Kent 2011) is another factor that undermines the power of the Law on Prevention of Domestic Violence and the Protection of the Victims.

It would be all too easy to overlook the violence. Family members might respond that everything is *tʰoammeaʔdaa*, meaning ‘that’s the habit’, but this is mistranslated as ‘things are normal’ and, in the next step, the euphemistic response *mɨn sǝv sok tei*, which is literally translated as ‘not very happy’ could really mean that there was violence in the family. Interviewers need to see past the demonstrative pronouns such as ‘that thing’ (*?aa rɨəŋ nuh*) and ‘I don’t really know much about this matter’ (*kɲom mɨn səv dəŋ tee pii rɨəŋ nəŋ*). There are subtle clues in the use of euphemisms: for example, extramarital sex being described as ‘*?aa rɨəŋ pon baat day*, which literally means ‘the thing that is the same size as the palm of the hand’, an allusion to the vulva of the woman that interfered with the marriage. The saying ‘Don’t tear open your chest lest the crow gnaw you’ (*kom haek truuŋ,?aoy k?aek cak*) warns against probing for details of family violence, which people feel should remain secret. Gossip spreads fast, as in the expression, ‘One crow [becomes] ten crows’ (*k?aek muey [tɨv cie] k?aek dɑp*. The crow is an enemy, a symbol of gossip, especially in the context of conflict in sexual and marital relations.

## Conclusion

Violence against women is usually seen as the product of economic, political, social, geographic, legal, historical and psychological forces. Insisting on the imposition of norm, feminist or social constructionist theories without making room for the *emic* perspectives of violence could be risky. Of course, these cultural constructions are moving, not essentialist and static.

The article in no way condones GBV. From a feminist perspective, power culturally reinforces itself and so, from that perspective, the cultural epigenesis of GBV could be said to arise from and reflect the problem.

There is a need for interventions to work with tradition and culture rather than only highlight it in problematic terms. To be clear, this article has presented the *perceived* causes and, as I argue, these underpin the ‘actual ones’. These insights are essential, even if they appear to be backward or nonsensical to the western observer—for the people who are affected a perceived cause is as good as a real one. Rather than foisting ‘foreign’ ideas on people, change has to harness rather than eradicate the traditional currents of violence that flow in the cultural stream. From a local perspective that continues to be held by gatekeepers such as Buddhist monks and, it would seem, by a significant proportion of the local population, its epigenesis is shaped by the guy ropes and pegs that connect the cultural attractors and pull the rivulets in the epigenetic landscape towards one ‘cultural attractor’ after another. It starts with an aggressive bedrock laid down in blighted endowment and continues as seeds of character, emerging as astrological forces. It erupts as the ‘Triple Poisons’, develops as the embarking by the perpetrator on the road to ruin. It sets the stage for the act of violence as the perpetrator’s mind becomes confused and seals his fate with the onset of moral blindness and an absence of shame and blame. This is a new way of looking at familiar concepts. We see in them metaphoric images such as heat, flame, fire and blood, colours like blacks and reds, and water boiling and bubbling and leading to violence.

The findings show just how deeply in the popular imagination the cultural pegs lie, and there is no doubt that violence permeates Cambodian culture, but this should not be read as suggesting that Cambodia is implicitly a culture of embedded violence. Perpetrators cannot hide behind an internal cultural logic that lends spurious purpose to their acts. A perpetrator may claim his atrocities were committed without free will, because he was ‘born with a birthmark’, for example, or ‘had unfortunate astrological configurations’ that lead to violence. The monks make plain that culture should *never* be used as a justification for violence against women.
